# Optimizing Magnetoencephalographic Imaging Estimation of Language Lateralization for Simpler Language Tasks

**DOI:** 10.3389/fnhum.2020.00105

**Published:** 2020-05-15

**Authors:** Leighton B. N. Hinkley, Elke De Witte, Megan Cahill-Thompson, Danielle Mizuiri, Coleman Garrett, Susanne Honma, Anne Findlay, Maria Luisa Gorno-Tempini, Phiroz Tarapore, Heidi E. Kirsch, Peter Mariën, John F. Houde, Mitchel Berger, Srikantan S. Nagarajan

**Affiliations:** ^1^Department of Radiology and Biomedical Imaging, University of California, San Francisco, San Francisco, CA, United States; ^2^Department of Neurological Surgery, University of California, San Francisco, San Francisco, CA, United States; ^3^Department of Neurology, University of California, San Francisco, San Francisco, CA, United States; ^4^Memory and Aging Center, University of California, San Francisco, San Francisco, CA, United States; ^5^Department of Neurology, Ziekenhuis Netwerk Antwerpen, Antwerp, Belguim; ^6^Department of Otolaryngology; University of California, San Francisco, San Francisco, CA, United States

**Keywords:** MEG, language lateralization, Wada, tumor patients, language tasks, picture naming, non-word repetition, verb generation

## Abstract

Magnetoencephalographic imaging (MEGI) offers a non-invasive alternative for defining preoperative language lateralization in neurosurgery patients. MEGI indeed can be used for accurate estimation of language lateralization with a complex language task – auditory verb generation. However, since language function may vary considerably in patients with focal lesions, it is important to optimize MEGI for estimation of language function with other simpler language tasks. The goal of this study was to optimize MEGI laterality analyses for two such simpler language tasks that can have compliance from those with impaired language function: a non-word repetition (NWR) task and a picture naming (PN) task. Language lateralization results for these two tasks were compared to the verb-generation (VG) task. MEGI reconstruction parameters (regions and time windows) for NWR and PN were first defined in a presurgical training cohort by benchmarking these against laterality indices for VG. Optimized time windows and regions of interest (ROIs) for NWR and PN were determined by examining oscillations in the beta band (12–30 Hz) a marker of neural activity known to be concordant with the VG laterality index (LI). For NWR, additional ROIs include areas MTG/ITG and for both NWR and PN, the postcentral gyrus was included in analyses. Optimal time windows for NWR were defined as 650–850 ms (stimulus-locked) and −350 to −150 ms (response-locked) and for PN −450 to −250 ms (response-locked). To verify the optimal parameters defined in our training cohort for NWR and PN, we examined an independent validation cohort (*n* = 30 for NWR, *n* = 28 for PN) and found high concordance between VG laterality and PN laterality (82%) and between VG laterality and NWR laterality (87%). Finally, in a test cohort (*n* = 8) that underwent both the intracarotid amobarbital procedure (IAP) test and MEG for VG, NWR, and PN, we identified excellent concordance (100%) with IAP for VG + NWR + PN composite LI, high concordance for PN alone (87.5%), and moderate concordance for NWR alone (66.7%). These findings provide task options for non-invasive language mapping with MEGI that can be calibrated for language abilities of individual patients. Results also demonstrate that more accurate estimates can be obtained by combining laterality estimates obtained from multiple tasks. MEGI

## Introduction

Hemispheric specialization for language is one of the most unique features of human cortical physiology. From a clinical perspective, being able to effectively isolate which hemisphere is “dominant” in an individual has high utility in presurgical planning for brain tumor patients and patients with medically refractory epilepsy. Formerly, the “gold-standard” approach for identifying hemispheric language dominance was the intracarotid amobarbital procedure (IAP) or Wada test, and remained the clinical standard for preoperative planning in the 1950s through 1990s (Wada, 1949). Unfortunately, the IAP test is a very invasive method with multiple associated risks and several complications (Simkins-Bullock, 2000; Hickok et al., 2008; Loddenkemper et al., 2008). These limitations lead to the adaptation of more non-invasive methods for language mapping including functional magnetic resonance imaging (fMRI) and magnetoencephalography (MEG) and have largely replaced the IAP for presurgical planning purposes (Papanicolaou et al., 2006). These neuroimaging measures identify lateralization by eliciting activations in the dominant hemisphere during expressive and receptive language tasks (Tharin and Golby, 2007; Ng et al., 2010). Unlike the IAP, these non-invasive mapping techniques can be easily repeated without risks (Tharin and Golby, 2007). While fMRI is the most frequently applied preoperative mapping technique, laterality results can be inconsistent as it fails to capture the fine temporal nuances of lateralization during speech reception and production (Tharin and Golby, 2007; Ng et al., 2010). In contrast, lateralization based measures using MEG imaging (MEGI) provide the high spatial and temporal resolution necessary to track the dynamics of language lateralization (Findlay et al., 2012). MEGI reconstructions during a verb generation language task MEGI has shown high sensitivity and specificity for language mapping (Hirata et al., 2004, 2010; Dalal et al., 2008; Kim and Chung, 2008). This is achieved by capturing and separating the stages of the speech reception and production pathway where both hemispheres are active (like primary auditory and motor cortex) from those lateralized processes that are typically anchored to regions of the dominant hemisphere in the temporal and frontal lobes (Hirata et al., 2004; Findlay et al., 2012; Hickok, 2012). As a result, language laterality indexes (LI) measured by MEGI have shown high concordance with IAP data and are now commonly used in presurgical planning (Hirata et al., 2010; Findlay et al., 2012).

The most validated and reliable task for non-invasive language mapping using fMRI and MEGI is the verb generation task (Price, 2010; Findlay et al., 2012), where subjects are instructed to generate a verb in response to an auditory stimulus presentation of a noun. As this task is complex, it requires the integration of perceptual (auditory), semantic, and phonological processes, lexical retrieval, and the planning, execution and monitoring of speech production (Kurland et al., 2014) and can be optimal for capturing both receptive and expressive processes generally lateralized for language function. However, given this complexity, this task can be challenging for patients with compromised cognitive and/or linguistic abilities, and its performance is difficult to score as many strategies can be taken by subjects to perform the task. Therefore, there is a dire need in non-invasive neuroimaging for other simpler language tasks to be available during pre-operative mapping that are easier to perform with analysis protocols tailored for each task. While other language tasks such as verbal fluency (Pirmoradi et al., 2016) and picture naming (Hinkley et al., 2016) have been administered during MEGI, these protocols have yet to be optimized as surrogate replacements for the IAP. As these tasks evoke different processes at different stages in time (compared to verb generation) optimized parameters for these datasets are critical to avoid incorrect interpretation of results, laterality reports and information provided to neurosurgeons (Brennan et al., 2016; Morrison et al., 2016). While the verb generation (VG) task has been optimized for MEGI (Findlay et al., 2012) no such procedure exists for other language tasks that may be easier for patients to handle. Additionally, while the predictive value of this procedure is high (∼90%) it is by no means perfect and could benefit from the addition of complementary linguistic protocols in case VG datasets are noisy, or source localizations from those datasets are poor.

To address this need for additional language mapping protocols in MEGI, we evaluated the utility of MEGI data collected during two linguistic tasks, non-word (or pseudoword) repetition (NWR) and picture naming (PN) and optimized these protocols by benchmarking them against VG laterality measures obtained in the same group of subjects. NWR and PN were chosen for both their simplicity and existing application during invasive clinical presurgical mapping procedures. Non-word repetition (NWR) is a fairly simple task that requires the coordination of a complex set of cognitive computations: auditory processing, information about the phonological sequence, articulatory processes and auditory feedback (Papoutsi et al., 2009). Although NWR does not rely on semantic processes involved in real-word repetition, NWR has proven to detect language structures during intraoperative language mapping (Sierpowska et al., 2016). PN is used as the gold standard technique for intraoperative language mapping (Duffau, 2007) and it demands at least the following processes: recognition of the visual stimulus as an instance of a familiar concept; access to the meaning of the word; access to the phonological word from (the learned pronunciation of the word) and motor programming and planning of articulation to say the word (DeLeon et al., 2007). Both of these tasks (NWR, PN) depend on processing within key lateralized regions of the speech and language network, including motor (posterior IFG, Broca’s area) and auditory (STG/MTG,/ITG) areas involved in speech perception and/or production (Price, 2010). This ability to tap into lateralized processes, combined with the fact that subject compliance for performing these tasks are high (even in compromised populations; Duffau et al., 2014), indicate that both NWR and PN have great potential for complementary or even surrogate non-invasive measures of language laterality.

Here, we validate measures of laterality in NWR and PN using a three-step process. First, we defined optimal time windows and regions of interest specialized in each task for laterality by examining patterns of beta (12–30 Hz) power decreases during NWR and PN in a training cohort (*n* = 30, *n* = 27) and compared these to results from VG protocols in the same patients. MEGI studies have demonstrated that cortical language areas in the frontal and temporal lobes show event-related power decreases in beta (13–25 Hz) and low gamma frequency bands (25–50 Hz), all thought to be a surrogate marker for cortical activation (Hirata et al., 2004, 2010; Kim and Chung, 2008). Specifically, we separated time periods in the response-locked (vocal onset) NWR and PN protocols and stimulus-locked (auditory presentation) NWR protocol (stimulus-locked PN was avoided as visual object recognition is not a process for language lateralization) in order to isolate time windows matching VG laterality values. Second, we validated NWR and PN analysis protocols by applying them to scans collected in a separate, comparable validation cohort (*n* = 30, *n* = 28). Finally, to see how well these results match against the IAP, we examine a small cohort with IAP data (*n* = 8) and the concordance of VG, NWR, and PN tasks with IAP results and examine composite estimates of LI from all tasks for determining hemispheric dominance for language.

## Subjects and Methods

### Subjects

To benchmark an existing preoperative MEG paradigm (using verb generation; Findlay et al., 2012) for language lateralization against other language tasks (non-word repetition, picture naming), we retrospectively analyzed the MEG data from a cohort of 96 subjects (40 females, 56 males) who underwent MEG language testing in the University of California at San Francisco (UCSF) Biomagnetic Imaging Laboratory (BIL) from 2013 to 2016. This cohort included 90 patients with brain tumors (50 right-sided, 40 left-sided) and 6 patients with medically refractory epilepsy (3 right-sided, 3 left-sided) who were surgical candidates for removal of the tumor or epileptogenic zone. Ages ranged from 12 to 75 years, with an average age of 42 years [standard deviation (SD), 14.6]. Thirteen were left-handed, and 83 were right-handed. Exclusion criteria included (1) if there was severe artifactual activity that could be noted in the MEG sensor array (peak-to-peak fluctuations in spontaneous activity > 2pT) and (2) if subjects were unable to perform the language tasks during practice.

All 96 patients had preoperative MEG for clinical purposes performing at least two different language tasks in the scanner: verb generation (VG) + non-word repetition (NWR) and/or picture naming (PN). In this large training cohort, 19 patients performed all 3 language tasks (VG + NWR + PN), 36 patients performed VG + PN and 41 patients performed VG + NWR. Consequently, the VG MEG data could be compared with NWR MEG data in 60 of the total 96 cases and with PN MEG data in 55 of the total 96 cases. To form our training cohort (which would be used to determine optimal parameters for NWR and PN, using VG as a base) we randomly selected half of the cases (30 for NWR and 27 for PN) from this pool of 96 cases.

In order to validate the lateralization parameters for NWR and PN generated from our analysis of the training cohort, we created a validation cohort from the other half (30 for NWR and 28 for PN) of the pool of 96 cases. In addition, we also retrospectively had access to a second cohort of 8 patients (2 females, 6 males) who underwent both preoperative MEG and IAP for clinical purposes. This IAP cohort was studied in order to test and see if the optimization parameters defined in the training cohort would produce lateralization results congruent with results derived from IAP. Time between IAP and MEG testing varied from a few months to 1 year. Six of these patients were brain tumor patients (4 left-sided, 2 right-sided) and performed all three language tasks (VG + NWR + PN) during MEG and 2 were epilepsy patients and performed VG and PN during MEG. Ages ranged from 22 to 51 years, with an average age of 39 years (SD, 12.3). They were all left-handed except for one patient who was right-handed. The same exclusion criteria as in the training cohort were applied. IAP results indicated language dominance in the left hemisphere for 5 and in the right hemisphere for 3. Demographic data (age, gender) was obtained in all participants and handedness was assessed via self-report. All subjects were informed and written consent was obtained for the study from all subjects. MEG studies were performed under a protocol approved by the UCSF Committee on Human Research (10-02027).

### IAP

The IAP was performed by a trained clinical neuropsychologist (D.A.C.-W.), based on an established IAP testing protocol (Loring et al., 1994). Amobarbital was introduced by hand over a 4–5 s interval into the internal carotid artery by using a transfemoral catheter. A single bolus injection was administered in most cases, and incremental injections were administered if marked hemiparesis was not induced. According to the Medical College of Georgia protocol (Loring et al., 1994) 100 mg of sodium amobarbital was typically chosen and amobarbital was administered to the side of suspected seizure onset first. Both left and right hemisphere injections were performed on the same day and there was a minimum of 30 min between injections. Language stimuli presented during IAP include items from the Western Aphasia Battery (WAB) and the Boston Diagnostic Aphasia Examination (BDAE). Language domains that were assessed include, comprehension via complex ideation and the token test, confrontation naming, repetition, reading, commands, and complex syntax. Language stimuli was individually tailored to the patient based on baseline abilities determined by testing prior to consenting the patient for the IAP. Specifically, language stimuli (comprehension, repetition) were presented during recovery period. Participants in the IAP sample were included if they underwent both IAP testing and MEG language testing for laterality, regardless of clinical case status, which produced a mixed sample of individuals with a variety of glioma location and refractory epilepsy.

### MEG

#### Data Acquisition

Magnetic fields were recorded in a shielded room using a whole-head MEG system (Omega 2000; CTF International Services LP, Coquitlam, BC, Canada) consisting of 275 axial gradiometers and 29 reference sensors used for computing synthetic third-order gradiometer measurements. The MEG signals were collected continuously and digitized at a sampling rate of 1200 Hz. Three fiducial coils (nasion, left/right preauricular) were placed to localize the position of the head relative to the sensor array. Head localization was performed at the beginning and ending of the collection to register head position and to measure head movement during the task.

#### Tasks

**FIGURE 1:**
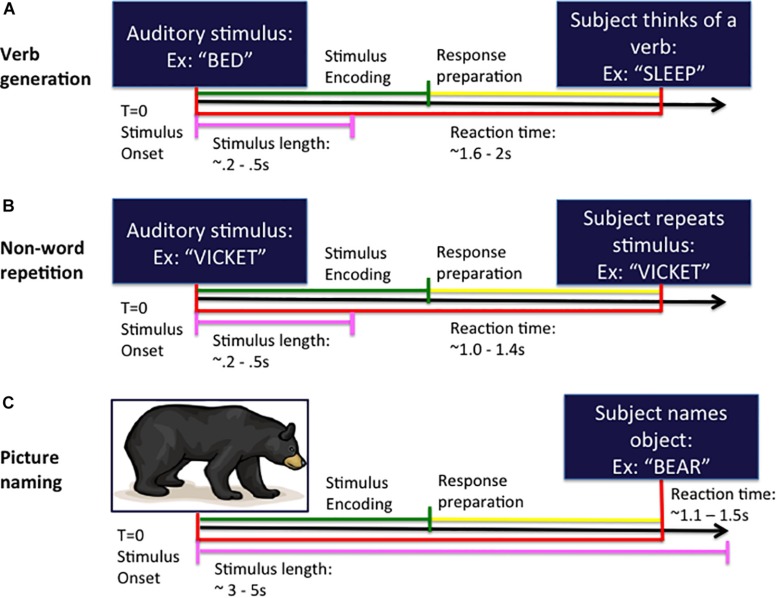
Task design for the verb generation **(A)**, non-word repetition **(B),** and picture naming **(C)** tasks. In each task, the subject is presented with either an auditory **(A,B)** or visual **(C)** stimulus at the beginning of the trial (time *T* = 0 ms) and responds by speaking into the microphone at response onset. Trials are organized into stimulus encoding (in green) and response preparation (in yellow) windowed segments prior to analysis.

The auditory verb generation task (VG) (Findlay et al., 2012; see Figure 1A) consisted of 100 nouns presented at a comfortable volume through earphones every 4 s. Subjects were instructed to think of a verb or “action word” associated with the noun presented at the beginning of the trial and to speak the verb into a MEG-compatible microphone.

The auditory NWR task (see Figure 1B) consisted of 100 non-words derived from the aforementioned set of VG nouns using letter substitutions respecting English phonotactical rules. Subjects were instructed to repeat the non-word presented at the beginning of the trial and to speak the non-word into the microphone.

For the visual picture naming (PN) test (Kaplan et al., 1983; Hinkley et al., 2016; see Figure 1C) an image of an object is projected onto a screen (100 trials) and subjects are instructed to name the pictured object into the microphone.

Stimulus onset (auditory noun presentation for VG; auditory non-word presentation for NWR; visual picture presentation for PN) and vocal responses were digitized on separate analog-to-digital channels, marked through amplitude threshold detection, and verified by hand through visual inspection manually in each dataset.

#### Data Analysis

Before data analysis, both noisy MEG sensors and trials with either artifact (eye blink, EMG artifact, or other obvious sensor artifact exceeding 1 pT), no responses and false starts (vocal responses 300 ms before stimulus presentation) were removed from the datasets. Neural sources were spatiotemporally estimated from the MEG sensor data using an adaptive spatial filtering technique (Wipf et al., 2010). Datasets were reconfigured into stimulus-locked (auditory stimulus = 0 ms) and response-locked (onset of the vocal response = 0 ms) formats for separate analyses. Stimulus-locked analyses were not generated from the PN datasets as lateralized speech reception processes are not present during a visual object naming task. Spatiotemporal estimates of neural sources were generated using a time–frequency optimized adaptive spatial filtering technique implemented in the Neurodynamic Utility Toolbox for MEG (NUTMEG)^1^. This approach allowed us to observe non-phase-locked changes in brain activity, measured as either a significant negative or positive change in the modulation of oscillatory activity. A tomographic volume of source locations (voxels) was computed through an adaptive spatial filter that weights each location relative to the signal of the MEG sensors (Dalal et al., 2008). Lead fields (8 mm resolution) were generated using a multiple sphere head model (Lalancette et al., 2011) at the single subject level from a high resolution T1-weighted anatomical MRI obtained in each subject. Source power for each location was derived through a noise-corrected pseudo-F statistic expressed in logarithmic units (decibels) comparing signal magnitude during an “active” experimental time window versus a baseline “control” window (Robinson and Vrba, 1999). Experimental time windows during the stimulus-locked and response-locked periods were compared versus intertrial baseline resting window locked to 300 ms prior to stimulus onset. Stimulus-locked results ranged from 150 to 850 ms following auditory stimulus; response-locked results ranged from 850 ms before to 450 ms after onset of verbal response. While our time-frequency decomposition in source space overlaps speech onset in the response-locked analysis (at 0 ms), our analyses are focused on both the post-stimulus and pre-response periods to avoid any noise interference generated by the articulators during speech itself. We focus on source-space reconstructions in the beta (12–30 Hz) band given that suppression in this frequency range related to cortical activation in the left hemisphere is commonly observed in linguistic tasks (Hirata et al., 2004; Findlay et al., 2012; Wang et al., 2012) and is known to have high concordance with IAP data for language lateralization (Findlay et al., 2012). Data were passed through a 12–30 Hz filter bank and partitioned into partially overlapping time windows using broad windows (300, 100 ms step size) optimized for capturing spectral peaks in the MEG signal (Dalal et al., 2008). Anatomical MRIs were spatially normalized (standard MNI template, SPM8)^2^ with the resulting parameters being applied to each individual subject’s reconstruction through Nutmeg.

#### Task-Specific VOI Definition, Time Points, and Laterality Index

Laterality index (LI) for verb generation (VG) was derived from methods outlined in Findlay et al. (2012). Changes in beta oscillatory power were extracted from volumes of interest (VOIs) defined *a priori* for the purpose of LI estimation. Voxels within each spatially normalized time–frequency reconstruction were tagged with MNI labels corresponding to anatomical structure. Two large VOIs were created based on previous magnetoencephalographic imaging (MEGI) studies (Hirata et al., 2004; Findlay et al., 2012). VOI-TP (temporal-parietal speech areas) contained voxels labeled as superior temporal gyrus or supramarginal gyrus; VOI-F (frontal speech areas) contained voxels labeled as inferior frontal gyrus, middle frontal gyrus, or precentral gyrus (Findlay et al., 2012). LI was calculated by averaging across activation in the VOIs of the left and right hemisphere separately across the stimulus- and response-locked VG tasks. Under the assumption that greater beta-power decrease (a marker of functional activation) in one hemisphere versus the other was associated with stronger lateralization, we used the following formula: LI = −1 * (L − R)/(| L| + | R|), where L represents the averaged *F*-value in the left VOI and R represents the averaged *F*-value in the right VOI. These beta power changes are multiplied by −1 as we normally observe beta suppression (negative values) compared to inter-trial baseline as an index of neural activity (Findlay et al., 2012). An LI value of + 1 or −1 would indicate greater beta-power decrease in the left or right hemisphere, respectively. MEG was classified as right for LI < −0.1, left for LI > + 0.1 and bilateral (also known as “mixed dominance”) lateralization for −0.1 ≤ LI ≤ + 0.1. As validated in Findlay et al. (2012), LI during VG from the stimulus-locked analysis was derived from VOI-TP during the 650–850 ms time periods and from the response-locked analysis in VOI-F during the −850 to −650 ms time periods. Total LI was calculated by averaging each subject’s stimulus- and response-locked LIs.

We adapted this approach (Findlay et al., 2012) in order to calculate NWR and PN laterality. First, we generated task-specific VOIs for NWR and PN separately by selecting participants within the training cohort with strong left laterality (VG LI > 0.2) and averaging source-space reconstructions in the beta band for each task separately across all subjects within that cohort. The strong right lateralized group in the training cohort was too small (*n* < 3) and therefore not used. The stimulus-locked condition for the PN task was excluded from analysis as induced beta power change during that analysis only reconstructs bilateral visual processing and not lateralized receptive and expressive language function. We then defined VOIs specific to each condition (NWR stimulus-locked, NWR response-locked, PN response-locked) by only selecting regions in the anatomical atlas that were the most active during this group averaging of the left-lateralized participants. This included temporal-parietal regions only active during the stimulus-locked analysis of the NWR task in this strongly left lateralized sample, and only frontal regions active in the response-locked analysis of both the NWR and PN tasks in this sample.

Next, we defined the three most significant time points for NWR and PN laterality measures by extracting LI values from each task-specific VOI (separately for NWR stimulus, NWR response, PN response) and correlating this with each subject’s total LI (generated using pre-existing parameters for VG) within the training cohort (*n* = 30 for NWR, *n* = 27 for PN). The three consecutive time points with highest correlation with VG LI were then selected. Once these task- selective VOIs and time points were identified this way, total LI was calculated by averaging each subject’s stimulus- and response-locked LIs similarly as in Findlay et al. (2012).

Patients were then categorized based on LI for VG, NWR and PN into either left-hemisphere dominant (LI > 0.1), right-hemisphere dominant (LI < −0.1) or bilateral (or “mixed”: LI < 0.1 to LI > −0.1) laterality.

#### Reaction Times, Correlation, and Concordance

A 1 × 3 ANOVA (Bonferroni corrected) and *post hoc T*-tests were used to determine whether there are any significant differences in reaction times between the three language tasks. The correlation between the calculated VG, NWR and PN LI scores was measured with a Pearson correlation test.

All language lateralization protocols (for VG, NWR, PN) generated above in the training cohort were tested by being applied to a separate validation cohort (*n* = 30 for NWR, *n* = 28 for PN, pt31-60 for NWR, pt28-55 for PN) by comparing the NWR or PN laterality (left or bilateral or right) with the VG laterality in this cohort (left or bilateral or right). In addition, the IAP cohort (*n* = 8) was used to compare the MEG laterality (left or bilateral or right) with the IAP language results (left or bilateral or right). Concordance was defined when PN or NWR laterality (left or bilateral or right) perfectly matched laterality of VG (in both the training and validation cohorts) or IAP (small IAP cohort).

## Results

### Definition of VOIs for NWR and PN

**FIGURE 2:**
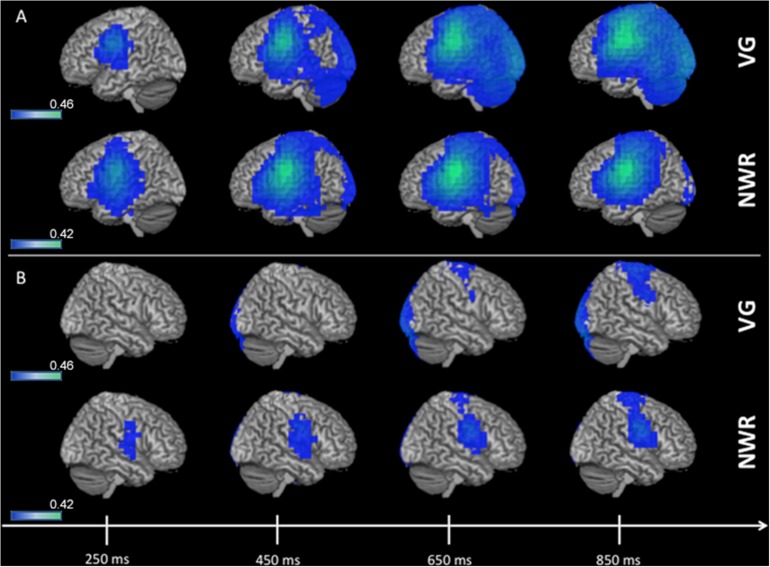
3D overlay of average changes (reduction of beta power, in blue) during stimulus encoding (stimulus-locked) for both the verb generation (VG) and non-word repetition (NWR) tasks in the strong left group. Following auditory presentation of the stimulus, reduced beta power is observable over temporal and parietal regions in the left hemisphere **(A)** and, to a lesser extent, over right frontal regions **(B)**. Individual beta power changes were spatially normalized to MNI space and averaged within each group for each condition. The group-averaged beta power changes were then thresholded at 25% of the absolute maximum *F*-value over the shown time course and displayed on 3D rendered brains in MNI space. Color bars are in pseudo-F values (in dB). Deep sources are not projected to the surface in this rendering.

**FIGURE 3:**
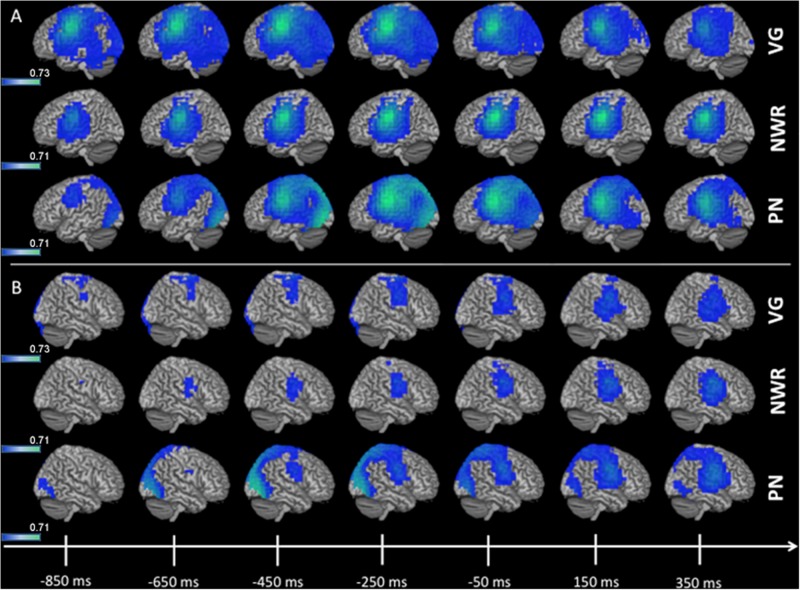
3D overlay of average changes (reduction of beta power, in blue) during response preparation (response-locked) for the verb generation (VG), non-word repetition (NWR), and picture naming (PN) tasks in the strong left group. Similar to stimulus encoding, during response preparation strong activation over temporal, parietal and frontal regions are observable in the left hemisphere **(A)** and to a lesser extent in the right hemisphere **(B)**. Conventions as in Figure 2.

Group (*n* = 96) averaged (grand mean, strong left group) changes in beta band (12–30 Hz) power for the stimulus-locked analyses (VOI-TP_VG_, VOI-TP_NWR_) are shown in Figure 2. For picture naming we do not generate a stimulus-locked analysis as we do not anticipate lateralized processing (with respect to language) for object recognition, as this process generally elicits activation bilaterally in higher-order sensory regions of cortex (see ventral posterior regions in Figure 3). For VOI-TP_VG_ (Figure 2A), decreases in beta power in the left hemisphere following auditory stimulation (auditory noun presentation, time = 0 ms) localized to the inferior frontal (peak MNI coordinate; *x* = −48, *y* = −3, *z* = 31), superior temporal (*x* = −48, *y* = −55.4, *z* = 12.7), and parietal cortices (*x* = −48, *y* = −20.9, *z* = 34), consistent with previous reports (Findlay et al., 2012; Hinkley et al., 2016). For VOI-TP_NWR_, similar decreases in beta power were observed in the left hemisphere over the IFG (*x* = −56, *y* = −3, *z* = 23) and STG (*x* = −56, *y* = −27.5, *z* = 11), but also over the middle temporal gyrus (MTG) (*x* = −56, *y* = −15.9, *z* = −3.8), and inferior temporal gyrus (*x* = −56, *y* = −13.5, *z* = −21.9), (ITG; Figure 2A). For both VOI-TP_VG_ and VOI-TP_NWR_ in the right hemisphere (Figure 2B), weak activation is observed over the IFG (*x* = 49.8, *y* = 4.6, *z* = 28.3) but not the STG, MTG or ITG in specific windows.

Group averaged (grand mean, strong left group) changes in beta band (12–30 Hz) power for the response-locked analyses (VOI-F_VG_, VOI-F_NWR_, VOI-F_PN_) are shown in Figure 3. For VOI-F_NWR_ and VOI-F_PN_, power decreases were found in frontal areas [inferior frontal gyrus IFG (*x* = −48, *y* = −2, *z* = 26.6), precentral gyrus PreCG (*x* = −48, *y* = −3, *z* = 31), middle frontal gyrus MFG (*x* = −48, *y* = 4.6, *z* = 38.2)] and also more posterior in the postcentral gyrus (PostCG) (*x* = −48, *y* = −24.2, *z* = 36.5) prior to and during speech production. Consequently, for VOI-F_NWR_ and VOI-F_PN_ the PostCG was added to the selection of VOIs (IFG, MFG, PreCG + PostCG for NWR and PN). This process created two novel VOIs specific to NWR (VOI-TP_NWR_ and VOI-F_ NWR_) and one novel VOI specific to PN (VOI-F_PN_) for further LI analysis.

We identified a main effect of condition type [*F*_(2, 219)_ = 111.7, *p* < 0.0001], with reaction times for VG (mean 1686 ms, *SD* = 327 ms) significantly longer in the large cohort (*n* = 96) when compared to either PN (mean = 1141 ms, *SD* = 178 ms, *t* = 12.2, *p* < 0.0001) and NWR (mean = 1115 ms, *SD* = 208 ms, *t* = 13.2, *p* < 0.0001).

### Training Cohort

#### Identification of Optimal Time Windows for LI in NWR, PN

**FIGURE 4:**
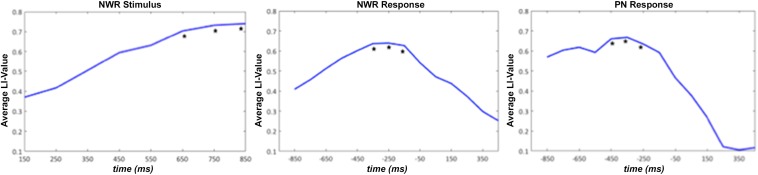
Average laterality index (LI) value for the strong left group for stimulus-locked NWR (time 0 ms = auditory stimulus presentation) and response-locked NWR and PN (time 0 ms = visual stimulus presentation). The three consecutive time points with the highest correlation with VG LI scores are indicated with a *.

In order to determine which time windows captured maximal lateralization for the NWR and PN tasks, we correlated (Pearson’s *r*) LI from each time window in NWR (VOI-TP_NWR_ in stimulus-locked, VOI-F_NWR_ response-locked) and PN (VOI-F_PN_ response-locked) with the overall LI derived from the VG task (Figure 4). Overall VG LI scores were calculated in the training group (*n* = 30 for NWR, *n* = 27 for PN) using the same method as in Findlay et al. (2012). For NWR and PN, pseudo-F values were extracted for each VOI and used to estimate LI for each time point. For VOI-TP_NWR_, the three consecutive time points with the highest correlation with VG LI scores were 650–850 ms (mean *r* = 0.32, Figure 4A) and for VOI-F_NWR_ −350 to −150 ms (mean *r* = 0.60, Figure 4B). For VOI-F_PN_, the three consecutive time points with the highest correlation with VG LI scores are −450 to −250 ms (mean *r* = 0.35, Figure 4C).

**FIGURE 5:**
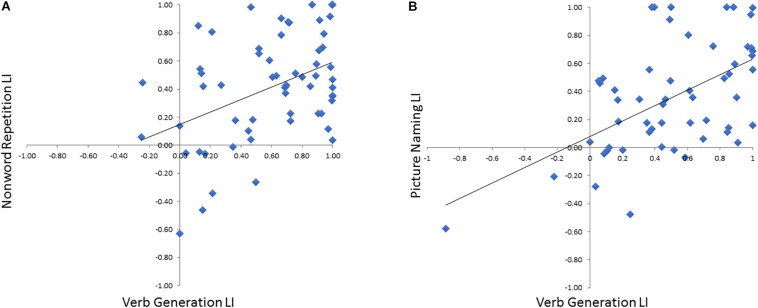
Correlations between VG LI for each subject with LI scores derived from NWR **(A)** and PN **(B)**.

To verify that the LI derived these optimal time windows we defined in the training cohort for both NWR (VOI-TP_NWR_: 650–850 ms, VOI-F_NWR_: −350 to −150 ms) and PN (VOI-F_PN_ −450 to −250 ms) accurately represented language laterality derived from the VG protocol, NWR-LI and PN-LI scores were separately correlated VG-LI scores (Figure 5). A medium correlation (*r* = 0.45, *p* = 0.010, *df* = 28) was found between VG LI and NWR LI (Figure 5A) and a strong correlation between VG LI and PN LI (*r* = 0.65, *p* < 0.0001, *df* = 25; Figure 5B).

### Validation Cohort

#### Concordance of Optimized NWR and PN Parameters for LI With VG LI

In the separate validation cohort (*n* = 30 for NWR, *n* = 28 for PN), laterality categories (left, right, bilateral) revealed that NWR laterality was concordant with VG laterality in 87% (26/30) of cases. PN laterality was concordant with VG laterality in 82% (23/28) of cases.

### Clinical Case Status

Lateralization indices broken down by clinical case status are shown in Table 1. Given that the data collected for this study was done for clinical pre-neurosurgical mapping efforts in the lab, considerable heterogeneity was seen across our sample with respect to clinical condition (tumor, epilepsy) as well as within each clinical condition (tumor location, hemisphere effected). Although our sample size is limited, there does not seem to be a significant relationship between clinical case status and reliability for using NWR and/or PN on evaluating laterality. Even in our largest groups (left and right frontal tumor) concordance between VG and NWR/PN was high (83–100%).

**TABLE 1:** Results broken down my clinical case status (epilepsy, tumor location), sample size, and matching with laterality results from verb generation.

		**Picture naming**		**NW Repetition**		**Composite**	
	**n**	**n/Match**	**Concordance**	**n/Match**	**Concordance**	**n/Match**	**Concordance**
Epilepsy	9	6/8	75%	1/1	100%	7/9	78%
Frontal: Left	17	9/9	100%	10/12	83%	16/17	94%
Frontal: Right	21	10/10	100%	12/13	92%	20/21	95%
Parietal: Left	4	3/3	100%	3/3	100%	4/4	100%
Parietal: Right	10	7/7	100%	6/6	100%	10/10	100%
Temporal: Left	10	7/7	100%	*5/6*	83%	10/10	100%
Temporal: Right	6	3/3	100%	3/3	100%	6/6	100%
Insular: Left	1	1/1	100%	1/1	100%	1/1	100%
Insular: Right	8	4/4	100%	6/6	100%	8/8	100%
Parietal-occipital: Right	2	*2/2*	100%	2/2	100%	2/2	100%
Parietal-temporal: Right	1	–	–	1/1	100%	1/1	100%
Frontal-parietal: Left	1	–	–	1/1	100%	1/1	100%
Frontal-temporal: Left	4	3/3	100%	3/3	100%	4/4	100%
Frontal-temporal: Right	2	–	–	2/2	100%	2/2	100%

### IAP Cohort

In the small IAP cohort (*n* = 8) hemispheric dominance for language as defined by IAP results and the three MEG tasks (VG, NWR, PN) is shown in Table 2. Two participants did not participate in the NWR task. There was full concordance (8/8) between results for VG and IAP in this cohort. For NWR, laterality was concordant in 4/6 subjects (66.67%) with the IAP, with two subjects with right IAP data (more right than left) evaluated as being bilateral using NWR LI. For PN, laterality was concordant with IAP in 7/8 (87.5%) subjects, with a lack of agreement between IAP and PN LI in a single subject. When LI scores for NWR and PN are averaged (NWR-PN Laterality, Table 2) concordance between these scores and IAP improve when NWR is used alone, with lack of agreement in only a single subject (Table 2). Maximal effectiveness is seen when LI scores for VG, NWR and PN are averaged into a composite score (Composite Laterality, Table 2) with these values showing full concordance with IAP data (8/8 subjects).

**TABLE 2:** Diagnosis, handedness and laterality results for IAP, individual MEGI and composite MEGI measures in the validation group.

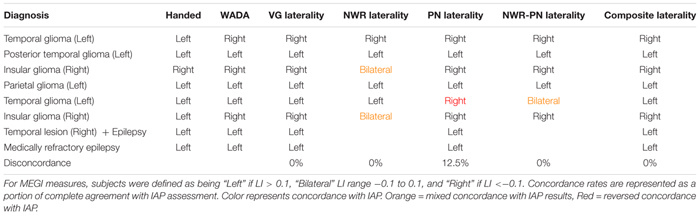

## Discussion

This study is the first investigation to use MEGI in order to evaluate the effectiveness of two simple yet novel tasks MEGI as measurements of language laterality in a large group of subjects (*n* = 60 for NWR, *n* = 55 for PN). Our findings demonstrate that hemispheric dominance for language can be reliably defined with MEGI using either NWR or PN – tasks which are easier for subjects to perform (demonstrated through significantly reduced RT), therefore ensuring strong task compliance. By benchmarking laterality during these tasks against a previously validated MEGI task for lateralization (VG), we identify which specific regions of the speech and language networks and which time points specific to each task accurately capture hemispheric lateralization for language. In our training cohort, regions functionally specialized for each condition at discrete time points during either NWR or PN reliably match with laterality ratings from VG. Testing this optimization through an independent validation cohort confirms high concordance between our protocols for NWR and PN with VG. High concordance between NWR/PN with VG is present across all clinical diagnosis categories (e.g., tumor location/epilepsy). Furthermore, in a cohort of individuals with IAP data, we identify good concordance between IAP and NWR and PN results when examined independently, and full concordance with IAP when a composite score is generated from all of these measures. Together, these findings not only provide a simpler option for pre-surgical mapping of language laterality for individuals with compromised linguistic abilities, but also illustrate the need for complementary task protocols for determining laterality and emphasize the power behind concatenating language tasks.

### Definition of Task-Specific VOIs and Time Points Optimized for NWR and PN Protocols

As verb generation reconstructions from MEG recordings have been previously validated as being highly concordant with IAP, we use this as our basis for optimizing protocols from the simpler tasks of NWR and PN. Since the VG MEGI protocol used in this study has a high sensitivity (100%) and specificity (92%) (Findlay et al., 2012) it acts as a reliable index for language laterality we can use to benchmark NWR/PN parameters. First, there was significant overlap between the language networks in the left hemisphere recruited during NWR and PN with the networks activated during VG for VOI definition (Figures 2, 3). This was expected as the three language tasks all share these cognitive processes: access to the phonological word form and programming, planning of articulation. However, there were some slight differences in activation patterns between the three tasks that required VOIs to be tailored to each analysis by the inclusion (or exclusion) of certain regions. These analyses illustrate the functional role each region plays in the separate tasks. In VOI-TP_NWR_, for example, areas MTG and ITG were added and for VOI-F_NWR_ the postcentral gyrus was added as beta power decreases were additionally shown in these regions. The involvement of more inferior temporal and more posterior (parietal) brain regions for NWR versus VG might be explained by the presentation of non-words versus real words. It is known that non-words rely more on short-term storage and attentional processes than real words and that inferior temporal and parietal regions are involved in these processes (Newman and Twieg, 2001; Sierpowska et al., 2016). In addition, numerous studies have shown that multiple peri-Sylvian regions are implicated in verbal repetition as repetition abilities arise from the coordination of a complex set of cognitive computations (auditory processing, phonological encoding, articulatory processes, auditory feedback) (Leonard et al., 2016). Therefore, repetition is a straightforward task for language localization, with high compliance. In particular, prior studies have successfully used NWR as a simple task for intraoperative language mapping (Sierpowska et al., 2016).

A separate pattern of task-specific VOIs were also identified for the PN analysis. The stimulus-locked VOI-TP_PN_ was not included in the lateralization paradigm as there was no receptive language component to this task, being a visual paradigm, beta power decreases in the stimulus-locked analysis were mainly found in the occipital and parietal regions bilaterally and not confined to a specific hemisphere. As a result, the MEG paradigm for PN only included the response-locked VOI-F_PN_ consisting of IFG, MFG, PreCG (regions recruited during VG, Figure 3) plus postcentral gyrus. Beta power decreases of parietal regions (such as postcentral gyrus) in PN has been shown in other MEG (Vihla et al., 2006) and ECoG studies (Edwards et al., 2010) as well. In addition, various intraoperative language mapping studies have used picture naming as a standard procedure because it is a straightforward, easy-to-administer task that involves a large language network including both frontal, temporal and parietal regions (in the ventral semantic stream and dorsal phonological stream) (Duffau et al., 2014). Collectively, across both of these tasks that are easy for the subjects to perform (NWR, PN) tailoring VOI selection on parts of the language network activated uniquely during each task strengthened our ability to localize this hemispheric specialization in MEGI.

Within each task-specific set of VOIs, specified points in time were found to be the most significantly correlated with VG laterality. Specifically, these were the 650–850 ms time windows post-stimulus presentation and −350 to −150 ms time window prior to speech onset for NWR, and the −450 to −250 ms time windows prior to speech onset in PN (Figure 4). Differences in time window selection between the three tasks may be due to different linguistic processes being involved depending on the language task. Whereas auditory stimuli were used for both the VG and NWR task (expressive and receptive component), visual stimuli were shown for the PN (expressive component). When comparing NWR with VG, attentional processes are highly more involved during NWR (Newman and Twieg, 2001; Sierpowska et al., 2016) but no semantic knowledge is needed during NWR. For PN visual recognition and access to the meaning are required (Vihla et al., 2006). In addition, the processing speed and reaction time required to complete the three tasks are different, given the inherent simplicity in the NWR and PN task protocols. Indeed, the reaction times for VG (mean = 1686 ms, *SD* = 392 ms) were significantly longer when compared to either PN (mean = 1141 ms, *SD* = 224 ms, *p* < 0.05) and NWR (mean = 1115 ms, *SD* = 198 ms, *p* < 0.01). Differing reaction times for the different tasks support the notion that each task requires different cognitive and linguistic processes, manifested through activation in different regions at different points in time. More expedient RTs for the novel tasks (NWR and PN, which were on the average 500 ms quicker than VG) demonstrate that these tasks are easier for participants to execute, and therefore may have more utility than VG for mapping hemispheric dominance in language compromised individuals.

### Concordance Between NWR/PN MEG Laterality Measures With VG

Application of our optimized VOIs and time windows (as established in our training cohort) when applied independently to our validation cohort replicate the finding that tailored spatial and temporal NWR/PN indices for laterality match well with VG, with concordance being high for both NWR and PN in this validation cohort, respectively 87 and 82%. As task selection may impact the interpretation of results (Brennan et al., 2016; Morrison et al., 2016), the calculated LI’s were compared to each other, with VG-LI used as a validated index of laterality (Figure 5). A stronger correlation between VG and PN LI’s (strong correlation: *r* = 0.65) was found than between VG and NWR LI’s (medium correlation: *r* = 0.45). Although the NWR task is presented in the auditory domain, cognitive and linguistic processing differs between this task and VG. Because the stimuli are non-words, no lexical trace exists in memory, and therefore the auditory non-word representation must be transformed directly into an articulatory representation without the mediation of existing vocabulary (Sierpowska et al., 2016), which is not the case for VG and PN. In summary, high concordance between NWR/PN with VG in an independent cohort using optimized NWR and PN laterality protocols indicate that measures from these more compliant tasks can be reliably used to estimate laterality.

To take this a step further, we explored NWR and PN as measures of language laterality in a separate smaller cohort where IAP data was available (Table 2). In this IAP cohort, high concordance was found for both NWR and PN with VG as well as results from IAP. The lower percentage for NWR is probably due to the low number (4/6) of which 2 out of 6 were bilateral instead of right. Given that none of these tasks (VG, NWR, PN) are fully concordant with laterality measures in both this study and other studies, it is reasonable to assume that a few points within this qualitative sample would not be fully concordant. Regardless, this consistent agreement between IAP, VG, NWR, and PN highlights the opportunity for tasks like NWR/PN to be used as surrogates for language laterality, especially in cases where subjects have difficulty executing more complex tasks like VG.

Rates of concordance reported here surpass those found in other imaging modalities, like fMRI, as the spatial and temporal precision inherent to MEGI reconstructions allow for more accurate targeting of hemispheric dominant processes. For PN (87% in validation cohort, 87.5% in IAP cohort) these findings are higher than what is generally found for fMRI, i.e., 81% according to the meta-analysis of Bauer et al. (2014). Reviewing the literature, concordance rates for fMRI and IAP have ranged from as low as 56% to as high as 100%. A variety of task paradigms, a diversity of methods to calculate concordance and the small sample sizes might explain the large range of numbers. Only a number of studies included more than 30 patients. In the study of Woermann et al. (2003) (*n* = 94) 91% concordance for covert word generation was found which is lower than our 100% for overt verb generation. Using a covert semantic decision task Benke et al. (2006) (*n* = 64) and Janecek et al. (2013) (*n* = 229) respectively reported 69–78 and 86% concordance. Therefore, even for MEGI protocols with less than perfect concordance with VG, validity for these tasks as surrogate measures of language laterality is quite high, particularly when compared to overt expressive fMRI studies.

Although both of our novel protocols (NWR, PN) did not have as high of a predictive value to measure laterality as VG, they can still act as reliable measurements of laterality, particularly in clinically affected populations where VG compliance might be challenging, and potentially moreso when combined together (Table 2). These results confirm many other studies showing that word retrieval tasks such as VG illustrate robust lateralization effects as they strongly activate both expressive and receptive language regions (Bowyer et al., 2005; Price, 2010; Pang et al., 2011; Findlay et al., 2012; Brennan et al., 2016). Regardless, our findings indicate that hemispheric dominance for language can still be gleaned from MEGI reconstructions using tasks that are easier to conduct. The potential for alternative tasks in mapping hemispheric dominance for language have been suggested before. A handful of previous studies have used picture naming to define LI in MEG studies (Shinshi et al., 2015; Hinkley et al., 2016). In case of severe naming problems (aphasia), NWR might be a good alternative. To the best of our knowledge, no MEG studies using NWR for laterality measures have been published. Other expressive tasks that have been used during MEG are verbal fluency (Pirmoradi et al., 2016) and reading (Pang et al., 2011) but they have not shown good agreement results. Several MEG studies applied receptive language (and memory) tasks such as word recognition tasks (Breier et al., 1999; Maestú et al., 2002; Papanicolaou et al., 2004; Doss et al., 2009; Pirmoradi et al., 2016) or semantic decision tasks (Tanaka et al., 2013) and reported concordance of 86–92% between MEG and IAP. However, as mostly left temporoparietal activations are found in these receptive MEG studies, information about the frontal expressive language regions is lacking.

While the goal of the present study was to establish lateralization parameters for NWR and PN, exploration of our composite LIs (averaging LIs of two or more tasks) demonstrated robust relationships with IAP results, particularly when these findings were mixed when using a single task. For example, in our IAP cohort, NWR protocols were unable to distinguish hemispheric dominance from bilateral organization in two participants. This became corrected in one subject when results from PN were included, and in both subjects when including results from all three tasks. Additionally, a single patient (left hemisphere glioma, Table 2) whose IAP identified left hemisphere dominance produced PN scores that were slightly low enough to fall within the range of right hemisphere dominance, likely due to a lack of fidelity from this single scan. Laterality scores in this patient did become concordant with IAP after inclusion of the VG and NWR scores, washing out this effect of a poor reconstruction during PN. Combination of NWR-PN together provided concordance in three subjects where individual measures were not fully concordant alone (Table 2). These findings suggest that, in the absence of a single reliable measure and/or in the presence of tasks that may be too difficult to execute for the participant (e.g., VG), composite laterality scores from two or more language tests may act as the most robust representation of hemispheric dominance for language. Based on this, we recommend the use of both PN and NWR for laterality measures (and their composite LI) if VG cannot be performed. Nonetheless, as even VG alone is not a perfectly concordant measure, the greatest power is derived from the composite (VG + NWR + PN) LI score, as even the verb generation task alone can be insufficient in establishing full specificity. MEGI data from each task can be collected quickly (∼8 min per run) and during a single session, reducing the dependence on results from complementary modalities (such as fMRI).

### Limitations and Future Directions

The overarching goal of the present study was to optimize parameters for determining hemispheric dominance for language in clinical presurgical cases. As a result, no attempt was made to control in our sample clinical case type (i.e., tumor, epilepsy) or impacted lobe/hemisphere. Although heterogeneity exists within our sample, high concordance (>75%) was identifiable across all groups regardless of these factors (Table 1). This is particularly true for combined laterality scores, as in the case of the second left temporal glioma patient (Table 2) who shifts from disconcordance to concordance once all scores are combined. It could be useful in the future to take factors like neurological diagnosis into account, as it is established that certain clinical conditions can push plasticity in language regions. In our own work, we have identified language lateralization “shifts” in glioma patients using VG following resection, related to initial magnitude of hemispheric dominance (Traut et al., 2019). Additionally, in epilepsy neuroimaging, epileptogenic foci location can often induce laterality shifts (Janszky et al., 2006; Rosenberger et al., 2009). Taking these features into account in the future could provide more tailored approaches for presurgical mapping, as it will become useful to a surgeon not only to avoid eloquent regions during surgery but be able to predict which regions will become eloquent post-resection.

Even though a substantial number of patients were used to evaluate the effectiveness of NWR and PN as measurements of language laterality (*n* = 60 and 55, respectively) MEGI, in our sample the number of patients that had IAP data available for confirmation was limited (*n* = 8). Many of these patients were left handed, and in the case of the single right-handed patient hemispheric dominance for language was atypical (although 5% of right-handed individuals are thought to be right language dominant; Knecht et al., 2000). In addition, it is difficult to discern if some trends we see in our IAP cohort (such as bilaterality in both right insular glioma patients with IAP for NWR) are significant trends with respect to clinical status, or outliers in the data. Nevertheless, it is valuable to report these findings from our smaller IAP cohort here, taking sample size into consideration. In order to fully confirm these tasks as surrogated for the IAP, it would require validation in a large cohort with both this procedure and all three tasks (VG, NWR, PN) in the same group of subjects, and effort outside the present scope of the study. Such an effort with a large sample size of IAP and MEG imaging patients would certainly be challenging. The use of the IAP has evolved and narrowed considerably in recent years as it is a very invasive method with multiple risks and limitations (Simkins-Bullock, 2000; Hickok et al., 2008; Loddenkemper et al., 2008), making it difficult to compare results from non-invasive neuroimaging studies with “gold standard” measures like the IAP. However, consistency with the IAP in the IAP cohort together with the high concordance results in the large validation cohort (>82% concordance with VG) are convincing enough to state that the developed VG, PN, and NWR MEG paradigms can be used to reliably estimate LI with MEGI.

In the future, it would be interesting to compare the MEG language data with intraoperative language mapping results. Direct electrical stimulation (DES) currently serves as the reference standard to define language localization in the context of brain surgery. While only the lesioned hemisphere (e.g., left) can be tested during DES, these data can confirm laterality measured by MEG if language errors are observed during stimulation. In addition, analysis of all intraoperative language sites found in a large cohort might enable more precise and specific regions that more accurately define language centers instead of the broader cortical regions that now correspond to the expressive and receptive VOIs. Furthermore, these results also need to be correlated with long-term functional language outcomes in the postoperative period in order to define predicting parameters for postoperative language impairments (language outcome). Finally, a comparison between preoperative LI and postoperative LI using different language tasks could provide more information on the effect of surgery on language lateralization and localization as well as brain plasticity mechanisms. Previous work in our group has identified shifts in language laterality following tumor resection using the verb generation task in MEGI (Traut et al., 2019). A major advantage of our expanded lateralization protocols (including NWR and PN, and potential composite measures) is that they are easy enough for subjects to engage in during the postoperative phase, even if some language imparments remain present post-surgery.

## Conclusion

To conclude, we are able to establish optimal parameters for novel MEGI data paradigms (NWR and PN) with high subject compliance by examining a large cohort of presurgical patients, and further demonstrate high reliability these laterality parameters in NWR/PN with independent MEGI and IAP cohorts. MEGIAs a result, MEGI using NWR and PN can potentially provide results consistent with the IAP and can be used to select cases for awake brain surgery with DES language mapping. MEG can act as a good alternative for fMRI, in particular for PN in cases of large tumors or vascular malformations, where the influence of the lesion on blood supply or metabolism can lead to distortion of the blood oxygenation level-dependent fMRI signal (Grummich et al., 2006). Demonstrating increased power through the combination of multiple tasks emphasizes the necessity for multiple (possibly even multi-modal) data sets in order to establish converging operations for identifying language laterality.

## Ethics Statement

Informed consent for the study was obtained from all subjects. MEG studies were performed under a protocol approved by the UCSF Committee on Human Research.

## Author Contributions

DM, CG, SH, HK, MB, PT, and AF helped collect data. ED, LH, and SN conceived and designed the analysis. M-CT contributed data or analysis tools. ED, M-CT, and LH performed the analysis. ED, LH, and M-GT, JH, PM, and SN wrote the manuscript.

## Conflict of Interest

The authors declare that the research was conducted in the absence of any commercial or financial relationships that could be construed as a potential conflict of interest.
